# Adaptation at the Syntax–Semantics Interface: Evidence From a Vernacular Structure

**DOI:** 10.1177/00238309231164972

**Published:** 2023-05-09

**Authors:** Frances Blanchette, Erin Flannery, Carrie Jackson, Paul Reed

**Affiliations:** The Pennsylvania State University, USA; The University of Alabama, USA

**Keywords:** Linguistic adaptation, syntax, American English vernacular, negative auxiliary inversion, negation, scope

## Abstract

Expanding on psycholinguistic research on linguistic adaptation, the phenomenon whereby speakers change how they comprehend or produce structures as a result of cumulative exposure to less frequent or unfamiliar linguistic structures, this study asked whether speakers can learn semantic and syntactic properties of the American English vernacular negative auxiliary inversion (NAI) structure (e.g., *didn’t everybody eat*, meaning “not everybody ate”) during the course of an experiment. Formal theoretical analyses of NAI informed the design of a task in which American English-speaking participants unfamiliar with this structure were exposed to NAI sentences in either semantically ambiguous or unambiguous contexts. Participants rapidly adapted to the interpretive properties of NAI, selecting responses similar to what would be expected of a native speaker after only limited exposure to semantically ambiguous input. On a separate ratings task, participants displayed knowledge of syntactic restrictions on NAI subject type, despite having no previous exposure. We discuss the results in the context of other experimental studies of adaptation and suggest the implementation of top-down strategies via analogy to other familiar structure types as possible explanations for the behaviors observed in this study. The study illustrates the value of integrating insights from formal theoretical research and psycholinguistic methods in research on adaptation and highlights the need for more interdisciplinary and cross-disciplinary work in both experimental and naturalistic contexts to understand this phenomenon.

## 1 Introduction

The question of whether and how exposure to unfamiliar or infrequent structures modulates speakers’ structural representations has been the subject of much psycholinguistic inquiry and debate (see Kaan & Chun, 2018 for a recent review). Studies addressing this question typically observe whether changes occur in participants’ behaviors during the course of an experiment, as a result of cumulative exposure to unfamiliar or infrequent structures. Researchers then draw inferences about grammatical changes that may underlie these observed changes in behavior. Such studies can serve as useful short-term analogs for linguistic processes that occur naturally over longer timespans, such as language acquisition and change (Kaan & Chun, 2018, p. 86), and can therefore inform fundamental questions, including how and why languages change over time, how people acquire languages, and what happens when languages and language varieties come into contact.

Studies have also employed unfamiliar vernacular structures as a way of examining structural changes during an experiment (e.g., Fraundorf & Jaeger, 2016; Kaschak & Glenberg, 2004). Observing the processing of unfamiliar vernacular structures is useful since these structures typically have properties that overlap with, but are not identical to, structures already familiar to speakers. The current study advances this line of research by asking whether speakers can learn semantic and syntactic properties of the vernacular negative auxiliary inversion (NAI) structure in English. The study moves beyond reading time data to focus on people’s interpretation of an unfamiliar structure, taking theoretical analyses of this structure as the basis for experiment design. It shows how consideration of theoretical models of hierarchical structure can advance our understanding of linguistic adaptation.

## 2 Background

### 2.1 Adaptation

In the psycholinguistic literature, *linguistic adaptation* is the phenomenon by which speakers and listeners change how they comprehend or produce linguistic structures as a result of cumulative exposure to similar structures in the input (see Kaan & Chun, 2018 for review). Such changes can occur at all linguistic levels, ranging from adaptation to a particular accent (i.e., at the level of phonology), or word choice, and even to particular syntactic structures. For instance, previous research has shown that people can adapt their interpretation of the quantities denoted by *many* and *some* to match the quantities presented during the course of an experiment (Yildirim et al., 2016; see e.g., Metzing & Brennan, 2003, for related findings at the lexical level). People can also rapidly learn to anticipate either a low- or high-attachment preference for ambiguous relative clause attachment, as in *The uncle of the girl who will ride the motorbike*, based on talker identity, and whether an individual talker produces sentences that always resolve to the high- or low-attachment interpretation (Kamide, 2012; see also Chun, 2018). People also tend to speed up their reading of less frequent syntactic structures (e.g., object relative and reduced relative clauses) through repeated exposure, reading them as fast as or even faster than related structures that are more frequent (e.g., Fine et al., 2013; Kaan et al., 2019; Wells et al., 2009; but see Harrington Stack et al., 2018, and Dempsey et al., 2020 for counterevidence).

In a related line of research, Luka and colleagues (Luka & Barsalou, 2005; Luka & Choi, 2012) investigated how reading aloud affects adaptation. These authors found that people rated grammatical but relatively infrequent pseudo-cleft sentences (e.g., *What the pharmacist recommended is to read the instructions*) as significantly more acceptable after reading sets of other pseudo-cleft sentences aloud, and that such modulations in acceptability could extend up to 48 hr post-exposure. However, there were no parallel changes in acceptability when participants were simply prompted to repeatedly make acceptability judgments about sentences without reading them aloud. Luka and colleagues argue that such results highlight the key role of “reading for comprehension” in linguistic adaptation.

Explanations for adaptation effects, such as changes in reading times and acceptability judgments, often implicate implicit learning mechanisms (e.g., Chang et al., 2006; Dell & Chang, 2014; Kleinschmidt & Jaeger, 2015 but see Reitter et al., 2011, for an alternative explanation). People are aware of the distributional frequencies of different linguistic structures in their input (e.g., Aslin & Newport, 2012; Yang, 2010). As the frequency with which one encounters a given structure changes—either over the short-term in an experimental context, or over longer periods of time in a more naturalistic environment—people adjust their expectations regarding the likelihood of encountering that same structure in the future. Over time, such adjustments can lead to cumulative changes in the linguistic system, which constitutes a form of learning. One key piece of evidence favoring such accounts is the inverse frequency effect, whereby adaptation over time is strongest for less common or unfamiliar, yet still attested, linguistic structures (e.g., Bernolet & Hartsuiker, 2010; Bock, 1986; Jaeger & Snider, 2013; Kaschak et al., 2011; Peter et al., 2015). In essence, adaptation parallels learning in that such effects are greater for something that is initially less well known versus something that is already well known. Furthermore, adaptation generalizes beyond the specific lexicalizations people encounter during initial exposure (see Mahowald et al., 2016 for review), providing additional evidence that cumulative changes over time occur at a more abstract level.

Researchers have also begun to explore adaptation by examining how people behave when exposed to an unfamiliar sentence type from a different language variety. Fraundorf and Jaeger (2016) showed that people unfamiliar with the English vernacular *needs* structure (e.g., *the car needs washed*, as used in the Midland dialect region; see Maher & Wood, 2011, for a review) will read such sentences as quickly as people familiar with the structure following exposure to as few as four to seven trials (see also Kaschak & Glenberg, 2004). Furthermore, participants then generalized this reading pattern to a different and unattested structure, which Fraundorf and Jaeger referred to as the *be*-drop structure (e.g., *The copier will be recycled because it no longer works*), reading *be*-drop sentences as fast as they read vernacular *needs* sentences. In contrast, participants who were already familiar with the vernacular *needs* structure exhibited slower reading times upon encountering *be*-drop sentences.

Fraundorf and Jaeger (2016) conclude that the generalization of reading times from vernacular *needs* to *be*-drop may be attributed to the idea that exposure to one unfamiliar structure leads participants to adapt their expectations about further unfamiliar structures in the subsequent input. Because participants who were previously familiar with vernacular *needs* had not treated these sentences as unfamiliar, they had not adapted their expectations about unfamiliar structures, hence their slower *be*-drop reading times. Fraundorf and Jaeger (2016, p. 45) further suggest that this type of adaptation of expectations may be restricted to structures that can be viewed as “similar.” Under this analysis, speakers who generalized their reading of vernacular *needs* to *be*-drop sentences would not have generalized to a structure like so-called positive anymore (Youmans, 1986; see Maher & McCoy, 2011 for a description and review), which the authors assume lacks structural similarities to vernacular *needs*.

Fraundorf and Jaeger (2016) do not discuss the theoretical linguistic implications of their assumption that vernacular *needs* and *be*-drop are similar. If we consider the assumption that these two sentence types are similar from a theoretical linguistic perspective, we see that it implies a form of phonological ellipsis of functional elements for both structures: *to be* is phonologically elided for vernacular *needs* (cf. *the car needs to be washed*), and *be* is phonologically elided for *be*-drop (cf. *the copier will be recycled*). Since this is a phonological and not a structural similarity, it is possible that participants’ faster reading times for *be*-drop reflected adaptation to the expectation that some elements of sentences would be deleted phonologically, as opposed to adjusting their expectations for novel structures. Considering further what this means for vernacular *needs*, the assumption is that the underlying structure is embedded infinitival passive (e.g., *the car needs to be washed, the baby needs to be fed*; but see Edelstein, 2014 for an alternative analysis). If this is the case, then vernacular *needs* is not a novel or unfamiliar structure *per se*, but rather a familiar structure with some functional elements phonologically elided. Participants’ faster reading times may therefore have resulted from their learning to map vernacular *needs* sentences onto structures already present in their grammars, as a type of analogical reasoning. The fact that participants had high accuracy on simple comprehension questions following the vernacular *needs* items that assumed synonymy with the embedded infinitival passive supports the idea that they were mapping this “novel” structure onto a structure already present in their grammar. As a preview to our results discussion, we will suggest analogical reasoning as a possible explanation for the adaptive behaviors observed in our experiment.

To date, a majority of research on syntactic adaptation has relied on the analysis of reading times and, to some extent, acceptability judgments. Far less research has considered how people’s interpretation of less frequent or potentially unfamiliar syntactic structures may change over time due to increased exposure (but see Chun, 2018; Kroczek & Gunter, 2017, for exceptions). Furthermore, in addition to questioning what readers actually adapt to, researchers have also asked to what extent adaptation effects, as reported in the experimental literature, are limited in scope to the specific experimental paradigm employed (see Kaan & Chun, 2018; Prasad & Linzen, 2019, for further discussion). The present study builds on previous work examining how people adapt to unfamiliar structures from another variety by investigating whether and how people learn to interpret NAI, a type of syntactic structure present in numerous vernacular English varieties. Extending beyond previous work on adaptation to unfamiliar structures from another dialect, the study design is crucially informed by theoretical analyses of the semantic and syntactic properties of this structure. The design and results show how consideration of the underlying hierarchical structure of the structures under investigation can inform experiment design in ways that help further our understanding of how and why speakers may change their behaviors in response to unfamiliar stimuli during the course of an experiment.

### 2.2 Negative auxiliary inversion

#### 2.2.1 General usage and interpretation

To understand the syntax of NAI, it is helpful to first observe negative yes–no questions such as the underlined portion of the following context:

1. A study group is discussing the main points from a lecture. One student is surprised to find that some classmates seem confused. She says: “Didn’t everybody understand what the professor said? I thought it was super clear.”

Example (1) contains a (negative) yes–no interrogative, realized syntactically by placement of the auxiliary in presubject position.^1^ Typically, such yes–no questions are realized with a final-rising intonation (Bolinger, 1978).

In many varieties of American English, strings that appear as yes–no questions as in (1) can also be used as declarative statements, as in the following context:

2. A study group is discussing the main points from a lecture. Most students agree that things were really clear, but one student disagrees. He says: “Didn’t everybody understand what the professor said. I was totally confused.”

In context (2), the underlined portion is string-identical to the interrogative in (1), with the negated auxiliary appearing in the presubject position. However, in this case, the string is pronounced and interpreted as a declarative, with a meaning equivalent to “not everybody understood what the professor said.” This is the phenomenon of NAI. At its core is the relationship between the subject and the auxiliary, which occur in an order that is non-canonical in both standardized and vernacular Englishes. Two further descriptive characteristics of NAI are that the auxiliary must be negated (e.g., Parrott, 2000; White-Sustaíta, 2010), and the negation appears as the clitic *n’t* as opposed to the marker *not* (Blanchette, 2015; Matyiku, 2017; Parrott, 2000; Salmon, 2018).

Labov et al. (1968) and Labov (1972) observed NAI use by vernacular African American and Latinx speakers in New York, and it has also been observed in White Alabama English (Feagin, 1979), West Texas English (Foreman, 1999, 2001; Matyiku, 2017), Vernacular Texas English (Salmon, 2018), African American English (Green, 2002, 2014; Parrott, 2000; Sells et al., 1996; Weldon, 1994), and Appalachian English (Montgomery, 2004; Montgomery & Hall, 2004; Tortora & den Dikken, 2010; Wolfram & Christian, 1976). An overview of the literature on NAI can be found in Matyiku (2011).

Note that the NAI example in (2) above contains the non-negative, universal quantifier subject *everybody*. In anticipation of our methods, we note that this particular NAI pattern served as the focus of our investigation. This is despite the fact that it may be less acceptable than other more frequent forms (e.g., Blanchette & Collins, 2018), and despite the fact that NAI most commonly occurs with morphologically negative subjects, as in *Didn’t nobody understand* (e.g., Blanchette & Collins, 2018; Matyiku, 2017; Sells et al., 1996). NAI structures with morphologically negative subjects can also be classified as Negative Concord structures, in which two or more syntactic negations contribute a single semantic negation (as in the “I ate nothing” reading of *I didn’t eat nothing*). Negative Concord is highly stigmatized in English and can occur independently of NAI. Because of this stigma, and because of the independent syntactic and semantic properties of sentences with two negations, it was necessary to avoid Negative Concord in our experiment. Since the Negative Concord form of NAI is relatively frequent and can be found in numerous popular culture references (e.g., the meme “Ain’t nobody got time for that”), this makes it more likely that participants would have been exposed to this form.^2^ Our focus on NAI sentences with universal quantifier subjects, though infrequent, therefore helped to ensure that participants had not been previously exposed to the structure, and further, it allowed us to isolate the semantic property of interest, namely, the wide scope of negation.

#### 2.2.2 Negation and quantifier scope

Theoretical analyses of NAI are built on important empirical generalizations about this structure type, one of which pertains to the phenomenon of taking scope. Since May (1977), scope-taking can be understood as the source of ambiguity in sentences like the following:

3. Everybody didn’t like the movie.

Sentence (3) is compatible with two truth-conditionally distinct interpretations: (i) everybody is such that they did not like the movie (i.e., nobody liked it), and (ii) not everybody liked the movie (but some may have). We henceforth call these the narrow-scope (i) and wide-scope (ii) negation readings.

The availability of both a wide-scope and a narrow-scope negation reading for sentences like (3) can be attributed to the presence of two scope-bearing elements: a negation (*n’t*), and a quantificational noun phrase (*everybody*). May (1977) proposes to model the phenomenon of scope-taking as an abstract syntactic movement of scope-bearing elements to a higher, structurally peripheral position. It follows that when two scope-bearing elements are present in a sentence, there are two possible abstract structures. The structures for (3) are illustrated here (QP = quantifier phrase, NEG = negation; irrelevant details omitted):

(3a) narrow-scope negation (3b) wide-scope negation

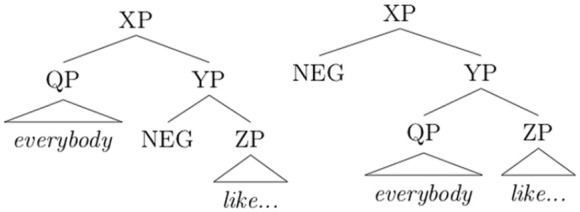


In structure (3a), the universal quantifier *everybody* takes wide scope, yielding the narrow-scope negation reading in which nobody liked the movie. Note that in terms of linear order, the string in (3) maps onto the structure in (3a). This contrasts with structure (3b), in which the negation takes scope over the quantificational subject yielding the wide-scope negation reading, despite its surface appearance following the quantifier. The fact that the wide-scope negation reading represented by (3b) is available for the string in (3) shows that quantifiers need not take scope in the order in which they appear on the surface.

Carden (1970, 1973) explored the extent to which individuals may prefer the wide-scope or the narrow-scope negation reading for sentences like (3), concluding based on context-free judgments that speakers have either a wide-scope negation dialect, a narrow-scope negation dialect, or a dialect that allows for both readings. In a study of related *all. . .not* structures (e.g., *all the moviegoers didn’t like the movie*) in the British National Corpus, Tottie and Neukom-Hermann (2010) find that the wide-scope negation reading is more frequent than the narrow-scope negation reading. For the purpose of this study, we assume that both the wide- and narrow-scope negation readings of (3) are generally available for English speakers, given the appropriate context.

The proposition in (3) can also be asserted in the form of an NAI structure, as follows:

4. Didn’t everybody like the movie.

Sentence (4) has the same two scope-taking elements as (3), *n’t* and *everybody*, so we might expect it to also be truth-conditionally ambiguous. However, as first observed in Foreman (1999, p. 11; see also Matyiku, 2017), in NAI only, the wide-scope negation reading is possible, and quantificational subjects must take narrow scope relative to the negation. This means that only (4b) is available as a reading of (4), and (4a) is not:

(4a) “Nobody liked the movie.”
(4b) “Not everybody liked the movie.” (wide-scope negation)

Numerous theoretical works have sought to model the lack of scope ambiguity in NAI (Blanchette & Collins, 2018; Foreman, 1999; Green, 2014; Matyiku, 2017; Sells et al., 1996; among others). Several theories derive the obligatory wide scope of negation by proposing that the negation raises over the subject overtly in the syntax. In theories such as Foreman (1999) and Matyiku (2017), the wide-scope negation property of NAI serves as the impetus for overt syntactic raising, while in Green (2014), the structure is associated with a special negative focus feature that triggers raising of the negation (see also White-Sustaíta, 2010).

Blanchette and Collins (2018) take a different approach to modeling the lack of scope ambiguity in NAI. They hypothesize that NAI involves the following grammatical constraint:

5. The NAI Subject Condition (Blanchette & Collins, 2018, p. 9, ex. 19))
  In NAI, the subject is negative.

For a sentence like (4), this means that the underlying structure of the quantifier phrase *everybody* is actually a negated quantifier phrase, akin to *not every player*. Instead of raising over the subject as in other theories, the negation instead raises from within the subject, as follows:

6. Blanchette and Collins (2018)

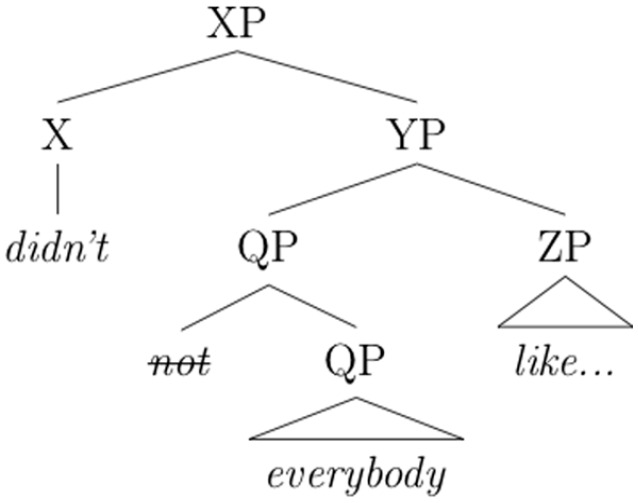


The negation enters the structure as a specifier of the quantificational noun phrase (see), and from there raises and cliticizes to a higher position (as -*n’t*). Given that the negation is introduced as part of the quantifier phrase, the structure captures the fact that there is no inverse scope reading in which the quantifier takes wide scope relative to the negation.

Note that prior to raising of the negation, the structure in (6) would yield the following string:

7. Not everybody liked the movie.

Thus, according to Blanchette and Collins (2018), the NAI structure in (6) is a structural analog of (7), with the minimal difference of a raised negation. As outlined in the next section, this proposal also captures restrictions on the type of noun phrase allowed to occur in the NAI subject position.

#### 2.2.3 Phrase type constraints on NAI subject position

In addition to the lack of scope ambiguity in NAI, previous theoretical work has also built on an important generalization regarding the type of subject that can occur in NAI, first observed by Foreman (1999, pp. 11–12; see also Matyiku, 2017 for an extensive description of the subject restrictions in NAI). Note first that NAI sentences with certain quantificational and Negative Polarity Item subjects are parallel in meaning and acceptability with sentences in which *not* occurs immediately preceding the subject:

8. Universal quantifier *every* subjects
(a) Didn’t everybody finish their homework. (Foreman, 1999, p. 11, ex. (29d))
(b) Not everybody finished their homework.
9. Quantificational *many* subjects
(a) Didn’t many people go to the party. (Foreman, 1999, p. 11, ex. (29b))
(b) Not many people went to the party.
10. Negative Polarity Item subjects
(a) Didn’t anybody seem to understand.^3^ (Feagin, 1979, p. 235, ex. (73))
(b) Not anybody seemed to understand.
11. Quantificational *half* subjects
(a) Didn’t half the students do their homework. (Foreman, 1999, p. 8, ex. (29f))
(b) Not half the students did their homework.

Importantly, the set of subject types that according to previous literature are impossible in NAI is also unacceptable when immediately preceded by *not*:

12. Referential noun phrase subjects
  (a) *Didn’t Jamie see the fight. (Matyiku, 2017, p. 16, ex. (1.19))
  (b) *Not Jamie saw the fight.
13. Definite noun phrase subjects
  (a) *Didn’t the teachers go to the party. (Foreman, 1999, p. 11, ex. (28c))
  (b) *Not the teachers went to the party.
14. Quantificational *few* subjects
  (a) *Didn’t few people live there then. (Matyiku, 2017, p. 75, ex. (3.5b))
  (b) *Not few people lived there then.
15. Quantificational *some* subjects
  (a) *Didn’t some person come. (Matyiku, 2017, p. 76, ex. (3.6b))
  (b) *Not some person came.

A quantitative acceptability judgment study with speakers familiar with NAI (primarily from Appalachia) in Blanchette and Collins (2018) confirms this pattern, noting that while the acceptability of attested NAI subjects as in (8) through (11) declines as a function of frequency, the unattested subjects in (12) through (15) are all equally unacceptable.^4^

Given these observations, speaker knowledge of NAI thus also appears to include an understanding of the type of phrase that can occur in the subject position. Different theories have different ways of deriving this knowledge. For example, Foreman (1999) (following Kiss, 1996), attributes the distribution of subjects to a mechanism of obligatory movement of referential subjects as in (12) through a Referential Phrase, which excludes structures such as (12a). Green (2014) appeals to a more general condition in which NAI subjects must be “strongly quantificational.”

For Blanchette and Collins (2018), the distribution of subjects in NAI is derived by the same mechanism that derives the obligatory wide-scope negation of NAI, namely, the constraint that NAI subjects must be (underlyingly) negative, as stated in (5) above. Because in this analysis the negation directly modifies the quantifier (see structure (6)), it follows that the same constraints which (dis)allow *not*-phrases in the subject position are also in effect in NAI. Under this theory, speaker knowledge of the constraints on NAI subject type is thus equivalent to speaker knowledge of the constraints on *not*-phrase subjects.

### 2.3 Adaptation at the syntax–semantics interface?

While the previous adaptation studies discussed above-examined reading times and acceptability judgments, in the present study, we use an interpretation task that builds on theoretical insights to probe whether and how native English speakers unfamiliar with NAI respond to its semantic and syntactic properties. A first task focused on NAI interpretation: We asked whether participants could adapt to the wide-scope interpretation of negation in NAI structures with universal quantifier subjects during the course of an experiment. During this interpretation task, participants were exposed to NAI sentences in ambiguous contexts and asked to choose between a wide- and a narrow-scope negation interpretation. A subset of participants received an additional “training” block that provided them with further exposure to NAI. For some of the participants who received the training block, the NAI structures were presented in contexts intended to unambiguously bias participants toward the wide-scope negation interpretation.

The design of the interpretation task was intended to allow us to investigate whether and how input impacts participants’ adaptation to the syntactic–semantic properties of an unfamiliar vernacular structure. If exposure leads participants to adapt, then this may be reflected in an increase in wide-scope negation interpretations later in the experiment. Unlike the vernacular *needs* structures employed in previous psycholinguistic studies (Fraundorf & Jaeger, 2016; Kaschak & Glenberg, 2004), NAI structures cannot be analyzed as phonologically elided variants of a more mainstream or supraregional structure. Instead, the surface position of the negation relative to a universal quantifier subject in NAI reflects a close and transparent relationship between structure and meaning. Capitalizing on this transparent relationship, we hypothesized that if participants adopt the wide-scope negation interpretation during the course of the experiment, then this can be interpreted as support for *structural* adaptation to an unfamiliar sentence type from another variety at the syntax–semantics interface. In other words, if participants shift toward the wide-scope negation reading of NAI during the course of the experiment, then this reflects a form of learning about the relationship between structure and meaning for this structure type. This learning may reflect genuine structural change, such that participants integrate the structure and corresponding meaning of NAI into their grammatical system, or it may be a more top-down, conscious decision-making process in response to the specific experimental task, which does not reflect any deeper structural change.

As with other adaptation research using short-term experiments, our experiment design does not allow us to observe whether and how changes in behavior reflect genuine changes to participants’ grammars (Dempsey et al., 2020). It does, however, allow us to observe whether speakers change their behaviors in any systematic fashion when encountering an unfamiliar linguistic structure from another dialect during an experiment. Since both top-down, conscious decision-making processes and structural change require the integration of new information with previous linguistic knowledge, then if systematic changes are observed toward a wide-scope negation interpretation of NAI, we can draw inferences about what aspects of participants’ previous linguistic knowledge would lead them toward this interpretation.

We further explored whether, beyond mere exposure, receiving unambiguous input that supports only a wide-scope negation reading would lead to better learning. As a preview to our results, because participants quickly adopted the wide-scope negation interpretation early in the experiment, our design did not ultimately allow us to fully determine the effects of ambiguous as compared with unambiguous input. We discuss possible reasons for these rapid changes in Section 5.

In addition to asking whether participants would adapt to the wide-scope negation interpretation of NAI structures with universal quantifier subjects, we also asked whether participants’ behaviors in the interpretation task would extend to performance on a subsequent naturalness rating task. The naturalness rating task included NAI structures with universal quantifier subjects presented in wide-scope and narrow-scope negation contexts. If exposure during the interpretation task leads participants to systematically select the wide-scope negation interpretation, and if this knowledge is generalized to a new task, then participants may give higher ratings to NAI items with universal quantifier (*every*) subjects in wide-scope negation contexts than in narrow-scope negation contexts. We also asked participants to rate NAI sentences with subject types to which they had not previously been exposed, with acceptable and attested *many* subjects and unacceptable and unattested *few* subjects (see Section 2.2.3). If participants rate NAI *few* sentences as less acceptable than NAI sentences with *many*, then this suggests they are extending their knowledge of the obligatory wide-scope negation interpretation of NAI to a wider range of subject types. However, if there is no evidence of generalization to the naturalness rating task—either in participants’ ratings of NAI items with universal quantifier (*every*) subjects or in their ratings of *many* versus *few* subjects—this would suggest that any adaptation exhibited in the interpretation task may be driven by top-down or task-specific strategies.

## 3 Methods

### 3.1 Participants

**Figure 1.**
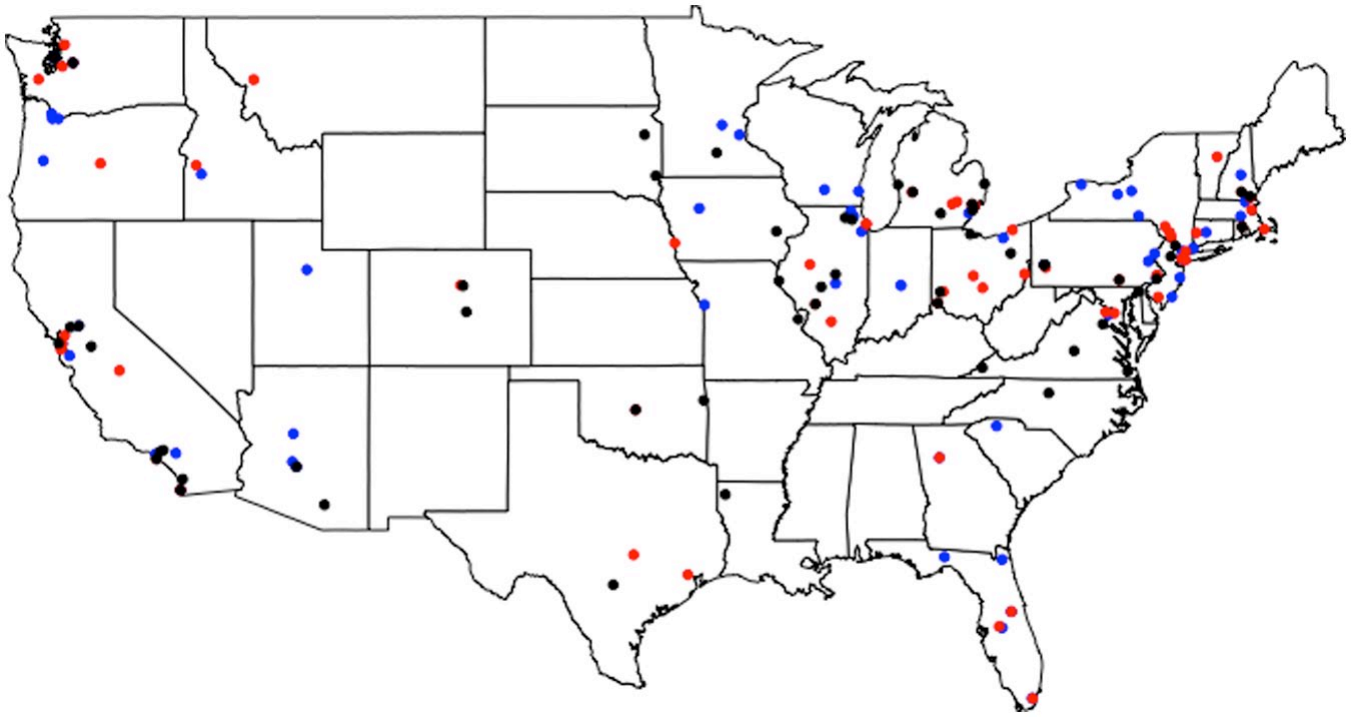
Locations where participants in each group reported to having spent the longest portion of their lives. Participants from the no additional training group are coded in black, participants from the unambiguous training group are coded in blue, and participants from the ambiguous training group are coded in red.

Two hundred thirty-seven adult native speakers of American English were recruited via Amazon Mechanical Turk and randomly assigned to one of three groups: (1) a no additional training group, (2) an unambiguous training group, and (3) an ambiguous training group. Participants received $10 as compensation for their participation. Fifty-eight participants were excluded based on responses to a posttask questionnaire, either because they reported having spent a significant portion of their lives in a region where NAI is known to be in use (*n* = 37; excluded regions included West Texas and states considered part of Southern Appalachia) because they reported hearing the structure regularly despite being from a region where it has not been documented (*n* = 12), or because they participated in more than one of our group surveys (*n* = 9), such that the final number of participants was 179.^5^ Of these included participants, 59 received no additional training on the NAI structure (42 male; 17 female; mean age 36.4, range 22–71), 61 received additional unambiguous input on NAI which biased interpretation toward wide-scope negation (40 male; 21 female; mean age 36.2, range 22–62), and 59 received additional but ambiguous input on NAI (32 male; 27 female; mean age 35.5, range 22–59). These groups are similar or greater in size to participant groups in other adaptation work (cf. Luka & Choi, 2012). Figure 1 illustrates the regions where participants in each group reported to spending the longest portion of their lives.

### 3.2 Materials

#### 3.2.1 The interpretation task

**Figure 2.**
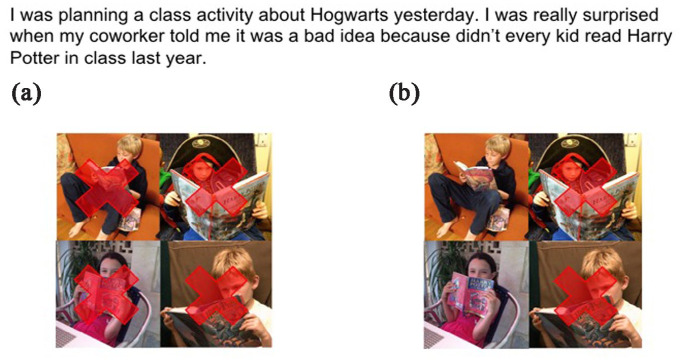
Sample ambiguous stimulus item from the interpretation task. Option (a) is narrow scope negation and (b) is wide scope negation. Option B is the wide-scope negation interpretation for NAI.

Within the interpretation task, participants were asked to select one of two images that best represented their interpretation of linguistic contexts including an NAI sentence with a universal quantifier subject (e.g., *everybody, every kid*). This task included 16 NAI sentences with an *every* subject placed in ambiguous contexts compatible with either a wide-scope negation or a narrow-scope negation interpretation (e.g., *I was planning a class activity about Hogwarts yesterday. I was really surprised when my coworker told me it was a bad idea because didn’t every kid read Harry Potter in class last year*). Eight ambiguous NAI sentences with *every* subjects appeared in the pretraining block and eight appeared in the posttraining block. The ambiguous contexts for these sentences were accompanied by a set of two images, one depicting a wide-scope negation interpretation, and the other depicting a narrow-scope negation interpretation. Visuals conveyed separate readings through the placement of red Xs over different objects in the composite image. To illustrate, Figure 2 contains the NAI structure *didn’t every kid read Harry Potter in class last* year presented in a linguistic context intended to be ambiguous between a wide- and a narrow-scope negation reading, followed by two graphics of students in class. One graphic has some but not all of the students Xed out, which is compatible with the wide-scope negation reading, and in the other graphic all of the students are Xed out, which is compatible with the narrow-scope negation reading. The location of the wide-scope negation versus narrow-scope negation interpretation was balanced across items such that both interpretations occurred as option A and option B the same number of times. In addition, all NAI structures were presented in embedded contexts (Green, 2014) to prevent participants from interpreting them as yes–no questions.

In between the pre- and posttraining blocks, the unambiguous training and ambiguous training groups both received a “training” block with 10 additional NAI sentences with *every* subjects. For the unambiguous training group, the training block included items presented in unambiguous contexts intended to bias the reader toward a wide-scope negation interpretation, as in (16). The ambiguous training group received the same NAI structures but in contexts that were ambiguous between a wide- and a narrow-scope negation interpretation, as in (17).

16. Wide-scope negation

I asked people to RSVP to my party by this Friday. I’m getting really frustrated because, even though I have a couple responses, didn’t everybody call me to RSVP.

17. Ambiguous

I asked people to RSVP to my party by this Friday. I’m getting really frustrated because didn’t everybody call me to RSVP.

The ambiguous training group thus received more exposure to the same type of items included in the pre- and posttraining blocks, while the unambiguous training group received implicit training toward the wide-scope negation interpretation. As in the pre- and posttraining blocks, all items in the training block were accompanied by images illustrating the narrow and wide-scope negation interpretations.

Thirty-two filler stimuli of three separate types were created to accompany the target stimuli. Sixteen items appeared in the pretraining block, 16 appeared in the posttraining block, and 10 appeared in the training block. The filler items included sentences with potentially ambiguous relative clause attachment (e.g., *My husband told me that the coach of the football player who was standing on the sidelines got really upset about the call by the referee*), vernacular forms (e.g., *Number 815 is running so fast that he might could win the race*, with double modal *might could*), or nonsystematic spelling errors (e.g., *The wresler in the middle won the gold medal*). The fillers were presented in context and with images, in a style consistent with the presentation of the critical items. Table 1 summarizes the design of the interpretation task.

**Table 1.** Summary of the Interpretation Task Design.

Group	Pretraining Block	Training Block	Posttraining Block
**Ambiguous training**	8 ambiguous NAIs	10 ambiguous NAIs	8 ambiguous NAIs
	16 fillers	10 fillers	16 fillers
**Unambiguous training**	8 ambiguous NAIs	10 unambiguous NAIs	8 ambiguous NAIs
	16 fillers	10 fillers	16 fillers
**No training**	8 ambiguous NAIs		8 ambiguous NAIs
	16 fillers		16 fillers

NAI: negative auxiliary inversion.

Twenty-two speakers from Appalachia and 24 speakers from regions where NAI has not been identified in the literature and who reported themselves as non-NAI users completed the interpretation task in a norming study prior to the present experiment (see Blanchette et al., 2020, for details). These norming data showed that speakers from Appalachia selected wide-scope negation responses more frequently (*M* = 0.67, *SD* = 0.25) than speakers from outside the region (*M* = 0.59; *SD* = 0.32), with the non-Appalachian speakers also exhibiting greater inter- and intraspeaker variability in their responses compared to the Appalachian speakers. Based on the Appalachian participants’ norming study responses, three critical items were modified prior to running the present study.

#### 3.2.2 The naturalness rating task

Naturalness rating stimuli were created by placing NAI structures with *every, many*, and *few* quantifier subject types in written contexts of one to three sentences. Participants were prompted to rate the naturalness of the NAI sentence as presented in context on a scale from 1 to 7 (1 = *completely unnatural*; 7 = *completely natural*). The task included eight sentences with *many* NAI subjects, as in example (18), and eight sentences with *few* NAI subjects, as in (19). Sixteen *every* NAI subject-type contexts were also included, eight of which biased participants toward a wide-scope negation reading, as in (20), and eight of which biased participants toward a narrow-scope negation reading, as in (21).

18. *Many*

The kennel was full of dogs who needed new homes. One family showed up last Friday afternoon to buy a puppy but for the most part did not many parents want a dog for their kids.

19. *Few*

I was arguing with Jen because she said she had blocked the most shots of any goalkeeper in the league. I had to break the news to her that did not few goalies block shots like she did.

20. Wide-scope negation context

Last night my coworkers and I decided to go out for karaoke. All the girls had a great time, and even though my friends Tom and Chris did a duet, I noticed that did not every guy sing a song.

21. Narrow-scope negation context

Last night my co-workers and I decided to go out for karaoke. All the girls had a great time, but I thought the guys did not want to be there because I noticed that did not every guy sing a song.

There were two versions of the 16 *every* subject-type items, such that each NAI sentence with a universal quantifier subject appeared in both a wide-scope and a narrow-scope negation context. Different versions of the same context were distributed to separate lists in a Latin Square design, so that each participant only saw one version of any given item.

Thirty-two filler items were also included with the naturalness rating stimuli. Eight filler items featured noncanonical word order (e.g., *My wife and I went on a trip to the Grand Canyon last weekend. It was amazing, but I forgot to bring a camera us with*), eight filler items featured different vernacular forms (e.g., *Grace was talking to her friend about whether to volunteer at the animal shelter. She knew she’d be busy on Monday, but she said she might could go Tuesday*), eight filler items featured an ambiguous relative clause attachment (e.g., *The judge at the recent murder trial was trying really hard to maintain a fair and impartial atmosphere in the courtroom. At one point during the trial the judge was annoyed that the attorney of the defendant who mumbled was questioned about personal matters*), while additional eight filler items contained no special features.

### 3.3 Procedure

Participants were recruited through Amazon Mechanical Turk and independently completed the survey on personal devices. All participants were prompted to complete the pretraining and posttraining modules, which had NAI sentences with universal quantifier (*every*) subjects in ambiguous contexts that elicited either a wide-scope negation or a narrow-scope negation reading. As described above, participants in the ambiguous and unambiguous training groups also completed a training module in between pre- and posttraining, either with *every* subjects in ambiguous contexts (the ambiguous training group) or in unambiguous contexts (the unambiguous training group). Participants completed the modules in one continuous task and were not made aware of these module changes. After the interpretation task, all participants completed the naturalness rating task in which they rated contexts featuring a variety of NAI subject types (see above). Target and filler items were presented together in randomized order within each task and task block.

Finally, participants completed a language background questionnaire, in which they self-reported personal and demographic information. The unambiguous training group and ambiguous training group surveys took approximately 60 min to complete, while the no training group survey took approximately 45 min to complete.

### 3.4 Statistical analyses

#### 3.4.1 The interpretation task

Analysis of the interpretation task results was conducted using a mixed-effect logistic regression model with the package lme4 version 1.1.26 (Bates et al., 2015) and *a priori* contrasts for hypothesis testing in R version 4.0.2 (R Core Team, 2020). Following methods outlined in Schad et al. (2020), contrast objects were assigned to a model matrix constructed using the MASS package (Venables & Ripley, 2002) and employed as fixed effects in the regression model. Testing block was included as a fixed effect, coded using repeated contrasts (.5 = *posttraining* vs. −.5 = *pretraining*). The training group was also included as a fixed effect and coded using Helmert contrasts. This allowed us to first compare the groups that received additional input with each other (1 = *unambiguous* vs. −1 = *ambiguous*, 0 = *no training*), and then compare the no-training group participants with the mean score of the two “training” groups combined (2 = no training vs. −1 = unambiguous and ambiguous). The random effects structure was determined according to a parsimonious approach (Bates et al., 2015; Matuschek et al., 2017), beginning with the maximal model including all random intercepts and slopes justified by the design, and removing components accounting for little or no variance whose removal did not lead to a loss in goodness of fit. The maximal model for the interpretation task included the fixed effects of block and training group, random intercepts for participant and item, a random slope for block by participant, and random slopes for the two training group comparisons by item—one for the contrast between unambiguous and ambiguous, and one for the contrast between no training and training. The stepwise model comparison led to the removal of random slopes for the two training group comparisons by item.

#### 3.4.2 The naturalness rating task

For the naturalness rating task, because the data were collected on a 7-point Likert-type scale, which is an ordinal as opposed to a continuous measure, the ratings were analyzed using ordinal regression (Liddell & Kruschke, 2018). This method differs from the common experimental syntax practice of using linear models (e.g., Sprouse & Almeida, 2017), which assume participants treat the rating scale as equally spaced. Unlike linear regression, ordinal regression allows for the possibility that participants will treat the distance between 1 and 2, for example, as larger than the distance between 2 and 3, because they are reluctant to give the minimum rating of 1. The inclusion of random intercepts and slopes for participant accounts for systematic differences in how participants treat the scale (thus obviating the need for z-score transformation), and random intercepts and slopes for item further account for potential systematic biases toward or against particular items within a condition.

A cumulative link mixed effects regression model was fit to the raw rating scores, using the *clmm()* function of the ordinal package (version 2020.8-22; Christensen, 2020) and a probit link function. Repeated contrasts, which compare adjacent levels of a factor, were used to code the context (i.e., syntactic subject type) factor. Since this factor has four levels, three comparisons were made: *many* versus *few* (.25 = *many, narrow-scope negation, and wide-scope negation*; −.75 = *few*), narrow-scope versus wide-scope negation (.75 = *narrow-scope negation*, −.25 = *wide-scope negation, many*, and *few*), and wide-scope negation versus *many* (*wide-scope and narrow-scope negation* = .5, *many* and *few* = −.5.^6^ The training group was coded using the same Helmert contrast coding as in the interpretation task analysis.

## 4 Results

### 4.1 The interpretation task results

The interpretation task included filler sentences with a potentially ambiguous relative clause attachment, a different vernacular form (e.g., a double modal), or an unsystematic spelling error. Participants’ accuracy levels for these items averaged between 90% and 91% in pretraining and between 89% and 90% in the posttraining block, indicating that they understood the nature of the task.

**Figure 3.**
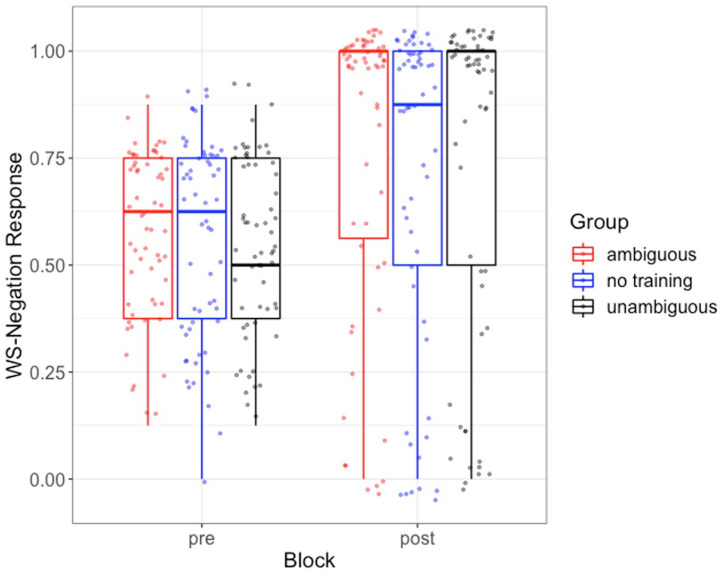
Proportion of wide-scope negation responses by group for pre- and posttraining blocks. Box plots show overall quartiles and median, and jittered points represent individual participants’ average target response rates.

Figure 3 shows results for the critical items, and Table 2 contains the results of the mixed logit model.

**Table 2.** Summary of the Mixed Logit Model for the Interpretation Task.

Predictor	Parameter estimates			
Fixed effects	Est.	Std. error	z-value	Pr (>|*z*|)
(Intercept)	2.05	0.30	6.83	<.001
Posttraining vs. pretraining block	3.54	0.54	6.61	<.001
Training group				
Input: unambiguous vs. ambiguous	−0.11	0.32	−0.35	.725
Training: no training vs. training	−0.11	0.18	−0.61	.544
Block × training group				
Block × group (input: unamb. vs. amb.)	−0.11	0.54	−0.20	.843
Block × group (training: no training vs. training)	−0.21	0.31	−0.69	.490
Random effects structure: (1|Item) + (1 + Block|Subject)				

As seen in Table 2, there was a reliable effect of block because all three groups were more accurate overall in posttraining (ambiguous: *M* = 75%, *SD* = 0.19; unambiguous: *M* = 75%, *SD* = 0.21; no training: *M* = 73%, *SD* = 0.16) than in pretraining (ambiguous: *M* = 55%, *SD* = 0.19; unambiguous: *M* = 56%, *SD* = 0.21; no training: *M* = 57%, *SD* = 0.16). However, there was no reliable block-by-group interaction because the accuracy gains made by the ambiguous and unambiguous groups were similar, and the overall gains for the two training groups were similar to those made by the no-training group.

#### 4.1.1 Rapid adaptation in the pretraining block

The fact that all three groups gave significantly more wide-scope negation responses in the post-training block than in the pretraining block, despite only two of the groups having received more input via a training block, suggests that many participants began systematically giving wide-scope negation responses at some point during pretraining. We visualized response patterns during each block to see how quickly this occurred, and to further explore whether the nature of the input during the training block impacted participants’ interpretation of the NAI items.

**Figure 4.**
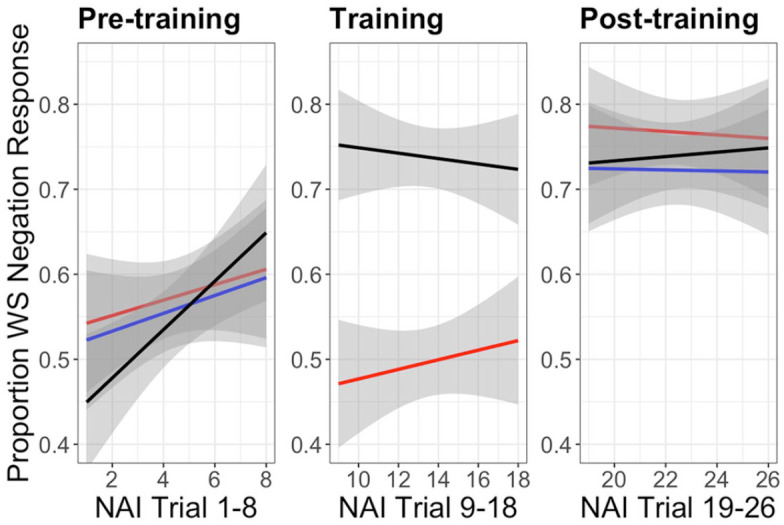
Proportion of wide-scope negation responses across trials for each group in each block. Smoothed curves and confidence intervals were generated using the geom_smooth function in *R* (method = “lm”).

As Figure 4 shows, all three groups progressed in a similar manner through the pretraining block. In fact, improvement to above chance occurs gradually over the entire block, and not immediately, for all groups. Among the unambiguous group, at the beginning of the training block, we see an immediate sharp increase in wide-scope negation responses which quickly stabilizes at a rate of around 75% for the rest of the task. In contrast, the ambiguous input group’s rate of wide-scope negation responses dips dramatically to just below chance during the first half of the training block. For this group, it is only after a greater amount of input that they begin to coalesce on the wide-scope negation interpretation again, jumping back up to around 75% during posttraining.

### 4.2 The naturalness rating task results

**Figure 5.**
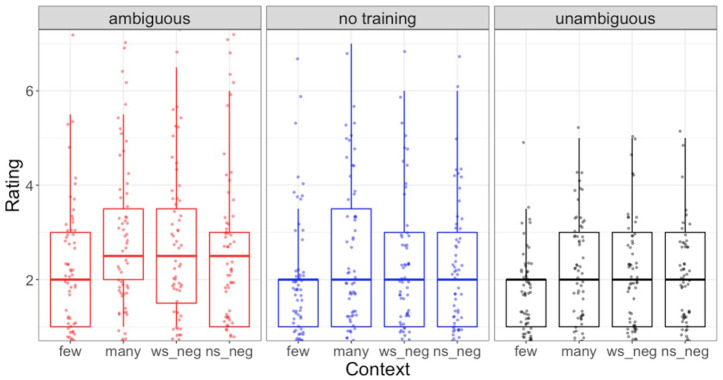
Naturalness ratings by group for narrow-scope negation (ns neg), wide-scope negation (ws neg), *many*, and *few* subject types. Box plots show overall quartiles and median, and jittered points represent individual participants’ median responses for each condition.

Figure 5 illustrates the results for the naturalness rating task. The results of the ordinal regression model are included in Table 3.

**Table 3.** Summary of the Ordinal Mixed Effects Regression Model for Naturalness Ratings.

Predictor	Parameter estimates			
Fixed Effects for Context	Est.	Std. error	z-value	Pr (>|*z*|)
Many vs. few	0.72	0.12	6.18	<.001
Narrow-scope vs. wide-scope negation	−0.08	0.05	1.54	.122
Wide-scope negation vs. many	−0.22	0.10	−2.29	.022
Fixed effects for group				
Input: unambiguous vs. ambiguous	−0.37	0.15	−2.37	.017
Training: no training vs. training	−0.03	0.09	−0.34	.734
Context × training group				
Many vs. few × no training vs. training	0.02	0.04	0.45	.653
Narrow vs. wide scope neg. × unamb. vs. ambig.	−0.02	0.05	−0.43	.666
Random effects structure: (1 + Group|Item) + (1 + Context|Subj)				

Within the context type fixed effects, there was a reliable effect of *many* versus *few*, because across groups participants gave higher ratings for acceptable and attested *many* items (*M* = 2.57, *SD* = 0.53) than for unacceptable and unattested *few* items (*M* = 2.02, *SD* = 0.74). However, there was no reliable effect of context for the *every* sentence, because, across groups, speakers did not rate *every* sentence in wide-scope negation contexts (*M* = 2.40, *SD* = 0.46) differently from those in narrow-scope negation contexts (*M* = 2.40, *SD* = 0.49). A reliable effect of wide-scope negation versus *many* reflects higher ratings for *many* items (*M* = 2.57, *SD* = 0.53) than for *every* item presented in wide-scope negation contexts.

For the group fixed effects, there was a reliable effect of input type during the training block, because the ambiguous training group (*M* = 2.68, *SD* = 1.52) gave higher ratings across all NAI item types than the unambiguous training group (*M* = 2.03, *SD* = 1.01). This was true specifically for the NAI items, since for the filler items the unambiguous group (*M* = 5.01, *SD* = 2.2) gave slightly higher ratings than the ambiguous group (*M* = 4.98, *SD* = 2.20). There was no reliable effect of input amount during the interpretation task, because the no training group (*M* = 2.32, *SD* = 1.38) gave ratings for NAI items that were similar to the two training groups combined. (This was also true for the no-training group’s ratings of the filler items (*M* = 4.95, *SD* = 2.18.) A lack of reliable interactions between context and training groups shows that there was no evidence that either input amount or input type during the interpretation task triggered larger differences between *many* and *few* items or between wide-scope and narrow-scope negation contexts.

## 5 Discussion

This study was designed to explore whether adaptation during an experiment can be observed at the interface between syntax and semantics, using insights from theoretical analyses of the vernacular NAI structure as a basis for investigation. The results of the interpretation task revealed a reliable increase in wide-scope negation interpretations during the course of the experiment, whereby the majority of participants initially selected both the wide- and narrow-scope interpretation, then shifted to systematically selecting the wide-scope negation reading early on during the pretraining block. In the naturalness rating task, participants from all three groups preferred NAI sentences with attested *many* subjects over those with unattested *few* subjects. We discuss these results here in light of both previous work on adaptation and theoretical models of NAI.

### 5.1 Adaptation in the interpretation task

With respect to the interpretation task, the present findings expand the scope of research on how exposure can rapidly modulate speakers’ interpretations of familiar, yet ambiguous, structures (e.g., Chun, 2018; Kamide, 2012; Kroczek & Gunter, 2017; Yildirim et al., 2016), by showing that speakers can coalesce on a particular interpretation, even when exposed to a structure that is completely unfamiliar. An open question in research on linguistic adaptation is the extent to which such adaptive behaviors reflect genuine changes in *structural* representations, or merely changes in sensitivity to atypical surface forms (e.g., Kaschak & Glenberg, 2004; see also Kaan & Chun, 2018). The results from the interpretation task suggest something beyond mere sensitivity changes can take place when participants encounter novel structures during an experiment. To achieve the observed change from chance-level performance in the pretraining block to reliably choosing the wide-scope negation interpretation in the post-training block, participants would have had to adjust the mapping between NAI structures and their meaning. Due to the nature of the task, which probed interpretation of an unfamiliar structure, this adjustment would have required some degree of analysis, whether explicit or implicit, beyond simply becoming more familiar with the surface form and using this increased familiarity to speed up processing. The study therefore moves beyond previous work investigating adaptation to the vernacular *needs* structure (Fraundorf & Jaeger, 2016; Kaschak & Glenberg, 2004), in which participants’ reading times reliably increased after only a small amount of exposure, by illustrating that speakers’ interpretation of an ambiguous structure from another dialect may also rapidly change after minimal exposure.

Although our results suggest some form of adaptation occurred during the interpretation task, as in previous laboratory-based studies exploring adaptation, there are important limits to what we can infer about the nature of the observed adaptive behaviors. In particular, it is difficult to know whether the behaviors are the result of genuine structural changes to participants’ linguistic systems, mirroring the types of changes that occur during exposure in natural conversation, or more surface-level, artificial changes that are limited to the experimental context. Given the nature of our interpretation task in particular, in which participants were given ample time to choose between two relatively transparent interpretations, it is possible that the observed changes were the reflection of a strictly top-down, metalinguistic decision-making process.

We would like to suggest that the results from the interpretation task are best understood as falling somewhere in between the two extremes of genuine structural change and surface-level, top-down decision-making. Since the pictures made the two logically possible interpretations clear and transparent for participants, they were forced to make a conscious decision between the wide-scope and narrow-scope negation reading. They began by providing both wide and narrow-scope negation responses, then converged on the wide-scope negation reading. Notably, they did this during the pretraining block, in which the NAI sentences were presented in ambiguous contexts.

Why might participants have shifted so quickly toward the wide-scope and not the narrow-scope negation reading? In the absence of evidence pointing toward a particular interpretation during the pretraining block, since the negation occurs first in the sentence, it may be that they simply chose the wide-scope negation reading because it is the more transparent “surface-scope” reading. This would be in line with routine-based theories of processing and language acquisition such as O’Grady (2013), in which the typical word order of a language triggers the development of a preferred order of interpretation in the presence of two or more scope-bearing elements. However, since these were adult native speakers of English, under O’Grady’s theory, we would expect their preference for wide-scope negation to be well established at the beginning of the task, and we would therefore not expect them to entertain the narrow-scope negation reading for any amount of time.

It is also possible that participants were analogizing to an already familiar structure, such as sentences with *not every* subjects, in which the negation also obligatorily takes wide scope (see Section 2.2). The idea that acquisition of novel structures may involve analogy to structures already present in an individual’s grammar is not new, and has long been employed in language acquisition research and usage-based theories (e.g., Bybee & Moder, 1983; see Behrens, 2017 for a review). In the context of our interpretation task, the hypothesis that participants were analogizing to *not every* structure would support theoretical analyses such as the one in Blanchette and Collins (2018), in which the wide-scope negation is derived because the negation directly modifies the subject underlyingly (see structure (6)). While our task design does not allow us to determine whether and at what level participants may have engaged in analogical reasoning, the results support the conclusion that participants were applying some aspect of their preexisting linguistic knowledge to the task when selecting the wide-scope negation reading.

With regard to the three-group design aspect of our experiment, in which we varied the type and amount of input participants were exposed to, we observed that during the interpretation task training block, the group that received unambiguous input appeared to reinforce the interpretation patterns they had already begun to establish when reading ambiguous input during pretraining. This resulted in consistently high rates of wide-scope negation interpretations throughout the training and posttraining blocks. For the group that received ambiguous input during both the pretraining and training blocks, on the other hand, it took longer for their rates of wide-scope negation interpretation to stabilize. This suggests that while ambiguous input can indeed lead to learning, the rate of learning may be slower and more variable than when unambiguous input is provided.

With respect to learning trajectories across the three groups during the interpretation task, one question that arises is why the group receiving ambiguous input throughout the entire experiment appears to drop back down to chance during the training block, after reaching higher levels of wide-scope negation responses during pretraining. One possibility is that, without unambiguous input to reinforce their initial intuition of how to interpret this unfamiliar structure, participants began to question themselves, either returning to chance levels or swaying in the other direction and reliably choosing the narrow-scope interpretation. However, if this were the case, then we might also expect to see the no training group’s rates of wide-scope negation response fall during the posttraining block, since they also received only ambiguous input. Instead, since the no training group’s rates of wide-scope negation responses remained high during posttraining, their lack of additional input appears to have supported (or at least not interfered with) their convergence on the wide-scope negation interpretation.

The design of the current study does not allow us to determine the nature of these differing behaviors across groups. Future studies of NAI interpretation employing online measures, such as eye-tracking, could provide more precise information on the trajectories participants follow across the experiment and within each block. Such studies would also provide insight into the precise linguistic cues participants exploit and their relative speed in employing these cues, which could serve to inform our understanding of the extent to which participants employed top-down, metalinguistic strategies in converging on the wide-scope negation reading.

Given the limitations of this and other laboratory-based experiments, future studies can also help us better understand the role and limits of task-specific, top-down strategies for adaptation, and to distinguish between these and more implicit types of learning in a laboratory-based setting. With respect to NAI learning specifically, one way to explore this would be to observe the effect of training input that biases participants toward the narrow-scope negation reading, as opposed to the naturally occurring wide-scope negation reading.^7^ If it is more difficult for people to learn an interpretation of NAI that differs from the true phenomenon, then this would support the conclusion that people were applying their preexisting linguistic knowledge in adapting to the wide-scope negation reading so quickly in the current study. Building on the current study, such future research would contribute toward our understanding of how unambiguous input facilitates learning of ambiguous structures from another dialect, and advance our understanding of how difference in the quantity and type of input shape trajectories of how people learn the meaning of NAI sentences.

### 5.2 Adaptation in the naturalness rating task

The naturalness rating task asked participants to focus on the acceptability of NAI sentences, some of which included a universal quantifier subject as in the interpretation task. Despite the fact that all three groups shifted toward the wide-scope negation interpretation during the interpretation task, this did not translate into meaningful differences in naturalness ratings for wide-scope versus narrow-scope negation readings with *every* subject sentences in the naturalness rating task. Similar to other laboratory-based research on structural priming and adaptation where exposure often consists of a short period of concentrated exposure to relatively homogeneous sentences, these results from the naturalness rating task could be taken to suggest that adaptation under more controlled circumstances may not generalize across distinct experimental tasks (e.g., Kaschak et al., 2011; see also discussion in Kaan & Chun, 2018).

The absence of evidence for more generalized adaptation to the wide-scope negation interpretation of NAI sentences with *every* subjects during the naturalness rating task may be due to differences in the type of knowledge that different tasks tap into. The naturalness rating task required participants to make distinctions based on acceptability rather than interpreting the unfamiliar structure itself. Participants may have focused primarily on the syntactic properties of the NAI sentences in the naturalness rating task rather than the presentation of these sentences in their larger context. However, to successfully make an acceptability distinction between wide-scope and narrow-scope negation *every* sentences, participants would have had to interpret these sentences in their larger context. The fact that participants did not do this robustly suggests that they were more focused on the syntactic properties of the target sentence than they were on computing its meaning in the larger context. This explanation is supported by the fact that, as Figure 5 shows, participants rated both the wide-scope and narrow-scope negation sentences with *every* subject higher overall than those with unnatural *few* subjects, while sentences with attested and natural *many* and *every* subjects received similar ratings. We therefore hypothesize that participants’ ratings were driven primarily by the syntactic properties of the subject as preceded by the negation, as opposed to the semantic interpretation of the sentence as elicited by the context.

In contrast with the lack of distinction between *every* sentence in wide- and narrow-scope negation contexts, participants did distinguish between NAI sentences with acceptable *many* and unacceptable *few* subjects. This occurred across groups, regardless of the amount or nature of exposure participants received during the training portion of the interpretation task, even though participants had likely never seen any of these structures previously. This suggests that at some level, they applied their previous linguistic knowledge of scope relations between negation and *many* versus *few*. As discussed in Section 2.2, with respect to NAI, as well as analogous *not* structures such as not many/*few people, differences between *many* and *few* sentences can be accounted for on the assumption that the negation directly modifies the quantifier in the syntax (Blanchette & Collins, 2018). It should be noted that since *few* also introduces an implicit negation and occurs immediately following an explicit negation, participants may have simply disliked the inclusion of two negations in proximity where a single affirmative (e.g., “Many people came”) would suffice. Such a possibility could be explored in a future study comparing how participants respond to unacceptable NAI structures with non-negative subjects (e.g., *Didn’t Jamie come.) Even if participants’ lower judgments of *few* sentences reflect a general dispreference for two negations in proximity as opposed to application of a specific syntactic constraint prohibiting their adjacency, this result still reflects application of preexisting linguistic knowledge. Thus, even if this is not evidence of adaptation or generalization from the interpretation task, it points toward another instance where participants were analogizing from their previously existing linguistic knowledge, extending this knowledge to novel NAI sentence types to complete an experimental task.

## 6 Conclusion

Using the vernacular NAI structure, the present study explored whether linguistic adaptation can be observed at the syntax–semantics interface. Participants reliably selected the wide-scope negation reading for NAI structures after only a brief period of exposure, and they readily distinguished between acceptable and attested sentences with *many* subjects and unacceptable and unattested sentences with *few* subjects in a separate naturalness rating task. This pattern of results was consistent regardless of the total amount or type of exposure to novel NAI sentences during training. As such, the present study showed ways in which linguistic adaptation research can extend beyond questions pertaining to reading times to investigate whether speakers can learn the semantic properties of an unfamiliar but grammatical structure from another dialect at the syntax–semantics interface. In so doing, this study provides evidence that speakers can rapidly change their interpretation of ambiguous structures from another dialect during the course of an experiment through analogical processes based on previously existing knowledge.

Our study design extended beyond existing research on adaptation to novel structures from a different dialect by integrating insights from formal theoretical research, and in particular, the syntactic properties of hierarchical structure and constituency. While future studies of syntactic adaptation may also benefit from considering these and other more fine-grained syntactic properties, we also acknowledge the limitations of investigating adaptation in the context of a short-term experimental study. More specifically, in the absence of any distinctions between wide- and narrow-scope NAI sentences with *every* subjects on the naturalness rating task, the possibility remains that any changes over time in the interpretation task were driven by the forced-choice design of the task itself. Further innovations and cross-disciplinary work are needed to draw stronger inferences about whether laboratory-based adaptive behaviors relate to the naturalistic linguistic adaptation that occurs in everyday interactions.

At the same time, even if task-specific top-down strategies were involved, we propose that the implementation of these strategies may have resulted from a form of analogy to a structure already present in their grammar. Analogy has played a central role not only in language acquisition and usage-based theories, but also in historical linguistics, and has been used to explain language change over longer periods of time (e.g., Lahiri, 2003). To our knowledge, however, it has not previously been considered in the context of linguistic adaptation.

Building on existing laboratory-based work, the present study adds to our understanding of how, through studying linguistic adaptation, we can gain insights into fundamental and cross-cutting questions in linguistics more broadly. By appealing to analogy, alongside syntactic theory, the present study highlights ways in which drawing on established concepts in other fields of linguistic inquiry can contribute to our understanding of the processes that may lead to adaptive behaviors. Though limited with respect to how they might extend to more naturalistic contexts, laboratory-based studies such as the one presented here provide a more controlled incubator to test for mechanisms that might underlie adaptation. It is important to note that, while what they reveal may be of a task-specific nature, the phenomena we observe in such laboratory-based studies are indeed connected to phenomena that exist in the real world, such as the acquisition of novel structures in a first language. This fact lends credence to the possibility that task-specific strategies observed here and in other similarly controlled environments may in fact reflect aspects of how linguistic adaption occurs in more naturalistic, everyday interactions.

## Notes

1. For a review and discussion of negative yes–no questions as in (1), see Dayal (2016, pp. 270–277). We set the meaning properties of these structures aside, as they are unrelated to NAI interpretation.

2. Thank you to an anonymous reviewer for pointing this out.

3. We maintain Feagin’s (1979) original spelling of the auxiliary, which reflects the vowel quality typically employed by the Alabama English speakers surveyed.

4. Salmon (2018) observes that for some Texan speakers, NAI sentences with definite subjects as in (13a) are possible under certain pragmatic conditions. We set this issue aside here, as it does not play a role in our experiment design or results interpretation.

5. Because speakers are not always aware if they use or hear a particular (vernacular) structure, we used region instead of reported exposure as a first pass in making our exclusions. However, since it is possible that surveys of the structure have missed certain demographics, we also took people’s self-reports of exposure into account. It is possible that this approach led us to exclude some people who had not been exposed, but this was necessary given the importance of lack of exposure for our study purposes.

6. The fact that these contrast settings yield the intended comparisons is not intuitive. The more intuitive settings for the *many* versus *few* comparison would be 1 = *many*, –1 = *few*, and 0 = *wide-and narrow-scope negation*; and for the wide-scope versus narrow-scope negation comparison they would be 1 = *wide-scope negation*, −1 = *narrow-scope negation*, and 0 = *many* and *few*. See Schad et al. (2020) for an explanation of the relationship between hypothesis testing and contrast matrices, and why repeated contrast settings do not intuitively reflect the hypotheses being tested.

7. Thank you to an anonymous reviewer for suggesting this.
